# Real world data and data science in medical research: present and future

**DOI:** 10.1007/s42081-022-00156-0

**Published:** 2022-04-13

**Authors:** Kanae Togo, Naohiro Yonemoto

**Affiliations:** Health and Value, Pfizer Japan Inc., Tokyo, Japan

**Keywords:** Real world data, Medical database, Observational study, Causal inference, Bias

## Abstract

Real world data (RWD) are generating greater interest in recent times despite being not new. There are various purposes of the RWD analytics in medical research as follows: effectiveness and safety of medical treatment, epidemiology such as incidence and prevalence of disease, burden of disease, quality of life and activity of daily living, medical costs, etc. The RWD research in medicine is a mixture of digital transformation, statistics or data science, public health, and regulatory science. Most of the articles describing the RWD or real-world evidence (RWE) in medical research cover only a portion of these specializations, which might lead to an incomplete understanding of the RWD. This article summarizes the overview and challenges of the RWD analysis in medical fields from methodological perspectives. As the first step for the RWD analysis, data source of the RWD should be comprehended. The progress of the RWD is closely related to the digitization, especially of medical administrative data and medical records. Second, the selection of appropriate statistical and epidemiological methods is highly critical for an RWD analysis than those for randomized clinical trials. This is because it contains greater varieties of bias, which should be controlled by balancing the underlying risk between treatment groups. Last, the future of the RWD is discussed in terms of overcoming limited data by proxy confounders, using unstructured text data, linking of multiple databases, using the RWD or RWE for a regulatory purpose, and evaluating values and new aspects in medical research brought by the RWD.

## Introduction

In the field of medical research, randomized clinical trials (RCTs) are the golden standard to estimate the causal inference between treatment interventions and outcomes. Real world data (RWD) are the data relating to patient health status and/or the delivery of the healthcare, routinely collected from a variety of sources regardless of the size of data (Hiramatsu et al., 2021; US Food & Drug Administration, 2018). Data from observational studies are also considered as RWD. The RWD are mutually complementary to limitations of RCTs, such as a small sample size of trials, participants with limited age groups including a smaller number of people, or no older people and minors, participants with limited complications, short follow-up duration, etc.

The RWD are generating greater interest in recent times although the RWD has been used since more than decades ago. Progress of the RWD is closely related to digitization, especially of medical administrative data and medical records. The UK established the first European electronic health records (EHR) database of Clinical Practice Research Datalink (CPRD) in 1987. In the US, the HITECH Act was enacted in 2009, providing funds toward encouraging medical practices to better adopt and make meaningful use of the EHR (Menachemi & Collum, 2011). In Japan, electronic claim of public health insurance was legalized in 1976, finally becoming an obligation in 2011. At present, the National Database of Health Insurance Claims and Specific Health Checkups of Japan (NDB) cover 99% of claims in Japan.

**Fig. 1:**
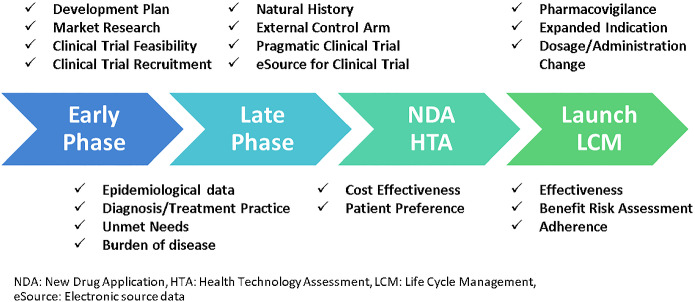
Use of real-world data during clinical development and post-launch in pharmaceutical industry

Various purposes of analysis using the RWD (hereafter, RWD analysis) in medical research include effectiveness and safety of medical treatment, epidemiology such as incidence and prevalence of disease, burden of disease, quality of life and activity of daily living, and medical costs. As an example of comparative effectiveness, long-term survival advantage among patients who underwent coronary-artery bypass grafting (CABG) was shown compared with percutaneous coronary intervention (PCI), using a large study population of 189,793 patients in total, from claims and patient registry databases (Weintraub et al., 2012). These days, the RWD and real-world evidence (RWE) are used for regulatory submission (Feinberg et al., 2020), as well as for other activities during clinical development and post-launch of drugs in the pharmaceutical industry (Fig. 1) (Togo et al., 2019).

The RWD in medicine is a mixture of digital transformation, statistics, data science, public health, reimbursement, pricing of products and regulatory science. Unfortunately, most of the articles describing the RWD in medical research cover only a portion of these specializations and that might lead to RWD’s incomplete understanding. Further, describing various sources of the RWD and relating analytic issues as biases (systematic errors) remain insufficient as well. Therefore, this article summarizes the overview of data source of the RWD and challenges of the RWD analysis in medical fields from methodological perspectives.

## Source of RWD

There are two approaches to collecting RWD: primary and secondary data collection (Schneeweiss & Patorno, 2021). Primary data are collected directly from study participants for the purposes of the study, and may be collected either retrospectively (via patient charts or other data sources), or prospectively (Mueller et al., 2018). Secondary data are obtained from existing health care data collection infrastructures, such as administrative claims databases, EHR databases, existing patient registries or study cohorts, or individual patient medical records.

Major data sources of secondary data are presented in Table 1 (Nabhan et al., 2019). Each database of secondary data has strengths and limitations. The overview of medical databases in Japan is annually surveyed by the Japanese Society for Pharmacoepidemiology and provided on the website (Pharmacoepidemiology & Database Taskforce, Japanese Society for Pharmacoepidemiology, 2021). The largest medical database in Japan is the National Database of Health Insurance Claims and Specific Health Checkups of Japan (NDB), which covers approximately 99% of health insurance claims in Japan. Although its use was limited to certain organizations for public welfare and academic institutions, NDB has been open to private companies for research of public health since October 2020 (Kaneyama et al., 2017; Kohsaka et al., 2021).

**Table 1:** Major data sources of secondary data (Nabhan et al., 2019)

Data source	Description
Administrative claims database	A health insurance claim is a request made for direct payment or reimbursement for medical services from hospitals, clinics, pharmacy. Claims data are systematic and well-structured. Large claims databases are available in many countries. However, claims are recorded to maximize the reimbursement and the data sometimes might be unrepresented as the disease name of clinical practice
EHR database (Evans, 2016)	An EHR is an individual patient health record. A typical EHR may include a patient’s medical history, diagnoses, treatment plans, immunization dates, allergies, radiology images, pharmacy records, and laboratory and test results. Although EHR databases are more likely to capture important health information about patients than administrative data, most of that information is unstructured
Patient registry	A patient registry is defined as an organized system that collects data and information on a group of people defined by a particular disease or condition, and that serves a pre-determined scientific, clinical and/or public health (policy) purpose (European Medical Agency, 2020). In addition to its use as secondary data, a registry-based study is another way of leveraging registry system. Registry often includes clinical outcomes, but missing data is common
Wearable, censor	Sensors and/or software apps on smartphones and tablets that can collect health‐related data remotely i.e., outside of the healthcare provider's office (Izmailova et al., 2018). These provide monitoring treatment response, including the monitoring of efficacy and safety of treatment, and monitoring of patient‐reported outcomes and/or quality of life measures

When selecting an appropriate data source for a research, protection of data privacy is one of the key elements, as well as strengths and limitations of each data source. The RWD includes medical data with sensitive personal information. Therefore, data privacy has to be protected for any types of data source in compliance with national data protection laws such as the US Health Insurance Portability and Accountability Act (HIPAA), the EU General Data Protection Regulation (GDPR), and the Japan Act on the Protection of Personal Information (Personal Information Protection Commission, Government of Japan, 2020; Office of Civil Rights, Department of Health and Human Services, 2002; The European Parliament & the Council of the European Union EUR-Lex, 2019; Wirth et al., 2021). In Japan, medical data is regarded as sensitive information and consent from patients (opt-in consent) is required to use such data for secondary purposes unless it is anonymized according to the act. However, for academic research, medical information regarding public health can be used with opt-out consent to provide patients an opportunity to refuse inclusion in the research (Ministry of Education, Culture, Sports, Science and Technology et al., 2021). Although database linkage enables large data creation, a wide range of personal information makes data linkage difficult, or at times, even impossible, because of data privacy.

## Statistical and epidemiological methods for the RWD analysis

Although the RWD can be leveraged for various research questions, the selection of appropriate statistical and epidemiological methods is highly critical than those for RCTs. In traditional clinical trials, randomization has long been an essential tool for minimizing bias by balancing underlying risk between treatment groups (Sherman et al., 2016). Among dozens of biases that have been defined, the major biases are the selection bias and the information bias (Table 2) (Rothman et al., 2008). A bias needs to be prevented by adequate designing of the study, since bias once identified, cannot be reverted. Confounding is another challenge in the RWD analysis. It can be controlled either in the study design or in its analysis.

**Table 2:** Biases in the RWD analysis

	Description	Example of limitation due to secondary data
Selection bias	Bias that results from procedures used to select participants and from factors that influence study participation	Insurance claims data, based on worker’s association, consists of relatively young and healthier people. The population of EHR depends on the types of hospitals (Rothman et al., 2008)
Information bias (Measurement error, misclassification)	Systematic error such as self-reporting or recall bias, measurement error bias, confirmation bias (investigator belief) (Althubaiti, 2016; Rothman et al., 2008). Immortal time bias is also a kind of information bias where immortal time is a span of time in the observation or follow-up period during which the study outcome could not have occurred (Suissa, 2008)	Diagnosis for reimbursement, validity of defined outcome, misclassification of drug exposure especially time-varying exposure due to limited data
Confounding	Confounding is related independently to both, the exposure and the outcome. It may create an apparent association or mask a real one (Strom et al., 2019). Confounding by indication can occur when participants, who undergo treatment, are more likely to have an underlying condition	Limited data of potential confounders

Basic principles to prevent a bias and confounding in design are random allocation (generally not applicable in RWD), subject selection or localization, stratification, and matching. Common epidemiological study designs using these principles are cohort studies of new-user design, nested case–control studies, and self-control studies (Table 3) (Strom et al., 2019). Furthermore, study designs for regulatory decision making in combination with clinical trials emerge (Table 3) (Baumfeld et al., 2020), whereas the purpose of those designs is to support the evidence from clinical trials rather than to prevent bias. The epidemiological study designs are adaptive to the studies for regulatory decision making as well.

**Table 3:** Study designs using the RWD (Baumfeld et al., 2020; Strom et al., 2019)

	Description	Limitation
Epidemiological study design		
Cohort studies of new-user design	Identifying patients who start a new drug and begin follow-up after initiation. Those patients have been evaluated by physicians who concluded that the patients might benefit from a newly prescribed drug. This makes treatment groups highly similar in characteristics that might not have been observed in the database	Secondary data requires the assumption that the first use of the drug after a certain period of no drug use is regarded as a new use
Nested case–control studies	Selecting all cases in the cohort, then randomly selecting one or more controls from risk-set for each case. The antecedent exposure is compared between cades and controls	Whenever the disease to be investigated is changed, controls need to be re-selected, as well as the cases. Whereas, the case–control study can easily consider many exposures
Self-control studies	This is a design where comparisons between exposures are made within subjects, thus significantly attenuating the problem of confounding	This design can only deal with an acute adverse event, a short wash-out period of exposure and requires precise data of exposure
Study designs for regulatory decision making in combination with clinical trials		
External control arm for clinical trials	Participants are selected from a cohort of RWD to be balanced with subject of a clinical trial in backgrounds for comparing key efficacy or safety outcomes	Differences in unmeasured confounders between the arms of clinical trial and the external control remain even after adjusting all measured confounders
Pragmatic studies	A randomized clinical trial incorporating pragmatic design elements. The intervention should be delivered as in clinical practice (Ford & Norrie, 2016)	This is the most feasible in health care systems with reliable and accessible electronic health records that capture the events of interest, which is at present are challenging in many countries
Long‐term follow‐up studies	Post‐marketing requirement studies of safety and effectiveness outcomes of interest require longer follow‐up durations	Regulators or companies may prefer RCTs due to feasibility (e.g., level of measurement and/or monitoring)

Statistical methods should be carefully selected in the RWD analysis for causal inference and estimation of treatment effect, as well as the study design. We summarize some statistical methods.

### Stratification, matching, and weighting using propensity score

Propensity score is the probability of treatment assignment (*Z* = 1 for treated, *Z* = 0 for control) conditional on measured baseline covariates (*X*) and the propensity score for subject *i* (*i* = 1, …, *n*) is $e_{i} = Pr\left( {Z_{i} = 1\left| {X_{i} } \right.} \right)$ (Rosenbaum & Rubin, 1983). The propensity score is often applied to stratification and matching to balance large numbers of covariates. However, the balancing between treatment groups using propensity score requires the assumption of no unobserved confounders which is rarely true in research using the RWD. Other difficulty is the distribution of propensity scores. It often does not overlap adequately between treatment groups when the treatment choice is strongly related with patient backgrounds.

Inverse probability weighting (IPW) using propensity score, or inverse probability of treatment weighting (IPTW) is an alternative method to estimate treatment effect with covariate adjustment. The inverse probability of treatment weight is defined as $w_{i} = Z_{i} /e_{i} + \left( {1 - Z_{i} } \right)/\left( {1 - e_{i} } \right)$. The weight can be highly large when a subject has very low propensity score. A number of alternatives are proposed to stabilize weights such as $w_{i} = {\text{Pr}}\left( {Z = 1} \right)Z_{i} /e_{i} + {\text{Pr}}\left( {Z = 0} \right)\left( {1 - Z_{i} } \right)/\left( {1 - e_{i} } \right),$ where ${\text{Pr}}\left( {Z = 1} \right)$ and ${\text{Pr}}\left( {Z = 0} \right)$ are the marginal probability of treatment and control in the overall sample (Austin & Stuart, 2015).

A large number of RWD studies with application of propensity have been published, but only a few reviews suggested the pitfall and the guidance (Austin, 2007; Yao et al., 2017; Zakrison et al., 2018).

### Structural models and doubly robustness

The difficulty of a RWD analysis, in addition to that caused by non-randomization, is bias due to time-varying exposures and confounders. These effects post-treatment initiation are not considered in Intent-to-Treat approach, which is generally employed in RCTs. Marginal structural models produce consistent estimates of the average causal treatment effects even in the presence of treatment changes, time-dependent confounders, and missing at random study dropout as application of inverse-probability weighting (Faries et al., 2014). Structural nested mean model is also applied to time-varying exposures and confounders, and are fitted using g-estimation. This model is better tailored for dealing with failure of the standard assumptions of no unmeasured confounders (Vansteelandt & Sjolander, 2016). Several applications of time-depending confounders have been reported in a wide range of diseases (Clare et al., 2019; Yang et al., 2014). Of these, one example is the comparative effectiveness study of angiotensin receptor blockers (ARBs), in patients with chronic heart failure (CHF), using a national cohort of beneficiaries of the US Department of Veterans Affairs medical care system (Desai et al., 2012). A marginal structural model in reducing mortality in CHF included the time-dependent confounder of hospitalization, which lies on the causal pathway from treatment to death. ARB treatment history and hospitalization were defined on monthly basis.

The idea of doubly robustness is to combine outcome and exposure modelling in a fashion that provides a valid estimate if either model is correct. For example, matching using propensity scores in a model for exposure, and then regressing outcomes on exposure and covariates in the matched sample. Robins et al. (1994) developed an improved augmented inverse probability weighting (AIPW) using the process of double robustness property involving 2 basic steps: first, fitting a propensity score model, and then fitting models that estimate the outcome $Y_{i}$ under treatment and control conditions, $f\left( {Z\left| {X_{i} } \right.} \right)$ (Kurz, 2021; Qi & Sun, 2014; Robins et al., 1994). The average treatment effect of AIPW can be estimated by

$$\frac{1}{n}\mathop \sum \limits_{i}^{{}} \left[ {\frac{{Z_{i} Y_{i} }}{{e_{i} }} - \frac{{Z_{i} - e_{i} }}{{e_{i} }}f\left( {1\left| {X_{i} } \right.} \right)} \right] - \frac{1}{n}\mathop \sum \limits_{i}^{{}} \left[ {\frac{{\left( {1 - Z_{i} } \right)Y_{i} }}{{1 - e_{i} }} - \frac{{Z_{i} - e_{i} }}{{e_{i} }}f\left( {0\left| {X_{i} } \right.} \right)} \right].$$

### Instrumental variable

Instrumental variable is the approach without assuming potential unobserved confounders. Instrumental variables naturally create quasi-random treatment choice and is related to the interested treatment. The instrumental variable estimator is simple: $\left( {E\left[ {Y\left| {T = 1} \right.} \right] - E\left[ {Y\left| {T = 0} \right.} \right]} \right)/\left( {E\left[ {Z\left| {T = 1} \right.} \right] - E\left[ {Z\left| {T = 0} \right.} \right]} \right)$, where T is the instrumental variable (Strom et al., 2019). An example of instrumental variable is site-level preference for the use of embolic protection devices (EPD) for assessing causality of EPD use during transcatheter aortic valve replacement (TAVR) on in-hospital stroke (Butala et al., 2021). However, the difficulty in finding a valid instrumental variable is the reason for its relatively limited use (Faries et al., 2014).

### Machine learning, AI

At times, the RWD is a large and high-dimensional data. Machine learning methods are used for identifying groups with disease prognostic or treatment response from a large data (Bakouny & Patt, 2021; Bica et al., 2021) In addition, machine learning and deep learning are leveraged for propensity score estimation. Application of AI to the RWD is an intensive research field to use complex RWD including texts, images, voice data, etc. Disease diagnosis from image data and outcomes, such as treatment response and adverse effects, extracted from physician notes are popular and practical themes using AI.

### Sensitivity analysis

Although it may be strange to deal with sensitivity analysis along with the methods mentioned above, it is worth referring to sensitivity analysis to understand the robustness of a study’s findings in a RWD analysis. Sensitivity analysis for quantifying a bias is sometimes called bias analysis. For unobserved confounders, external adjustment is to adjust relative risk using external evidence. For misclassification of disease or exposure, statistics can be adjusted using sensitivity and specificity estimated in a validation study or external literatures. For assessing the effect of a bias in the study design, definitions of baseline period, outcomes, and exposure can be varied in the sensitivity analysis (Rothman et al., 2008). Causal effects estimated in observational studies are not binary signals, with or without statistical significance, but are numerical quantities. To quantify the effect as unbiasedly and precisely as possible, multiple studies using the different RWD sources, and meta-analysis of them could provide a highly reliable estimate (Hernán, 2021). The practical implementation, and quite a few assumptions of the sensitivity and bias analysis are continuously discussed.

## Discussion

There are several approaches to overcome the limited data of a RWD database. Although administrative database has limited information, high dimensional propensity score approach can be exploited by applying proxy confounders of variables created from a large quantity of structured data, such as disease and prescription records (Bosco-Lévy et al., 2021). For the EHR, unstructured text data including physician’s progression notes and repots of test results is drawing attention. Clinical outcomes are derived from unstructured medical records using retrospective review and automated analysis, using natural language processing and AI. This is critical for the progress of comparative effectiveness research using the RWD. Linking multiple databases enables patient follow-up for a long period of time, or covers a wide range of personal information. The data privacy regulation in each country makes data linking difficult or at times, even impossible. As a countermeasure for medical research, in Japan, “Act on Anonymized Medical Data That Are Meant to Contribute to Research and Development in the Medical Field” (Next Generation Medical Infrastructure Law) was enforced in 2018 that allows certified enterprises to deal with identifiable medical information from multiple facilities.

In drug development, pharmaceutical companies and regulatory authorities consider utilizing an external control arm for a non-randomized clinical trial of a single arm when randomization may not be feasible or ethical (Nishioka et al., 2021; US Food & Drug Administration, 2018) Bias is greater problematic in comparison between treatment arms from clinical trial and the RWD, than the comparison between treatment arms within a database of the RWD. However, there is a strong need of the external control arm in rare disease area, and regulatory guidance has been issued in several countries. In Japan, the Ministry of Health, Labour, and Welfare (MHLW) and the Pharmaceuticals and Medical Devices Agency (PMDA) have been working to promote the RWD use for regulatory decision making. They have issued several guidelines, including the RWD use for post-marketing surveillance and registry data use for drug applications (Nishioka et al., 2021; Ishii et al., 2021).

The RWD provides new aspects on medical research. Research using the RWD provides results with an increased speed. It is based on larger data than research using primary data collection, with a relatively low cost, once the platform is established. Demands of rapid RWE in post-marketing safety surveillance are increasing considerably after the COVID-19 pandemic (Naidoo et al., 2021). For example, a study to explore the frequency and severity of myocarditis after COVID-19 vaccination used the data until May 24, 2021 from the database of Clalit Health Services in Israel, and the results were published in the New England Journal of Medicine just after about 4.5 months from the data period (Barda et al., 2021). Another aspect is new technologies as means of collecting the RWD novel outcomes created by digital devices, such as mobility and sleep by wristwatch type wearable and ECG by skin patch, yielding new values of drug effectiveness. These data from new technologies provide opportunities to apply new methodologies of data science dealing with large data of intensive longitudinal time periods (Izmailova et al., 2018).

There still exists large potential for improvement in the ways of demonstrating the reliability or degree of bias, and the uncertainty about the evidence obtained from the new types of RWD. We would rather quantify them for considering the effect on decision making, than emulating the inference of RCTs. Moreover, the RWD reflect “real world” of local regions, and it is important to understand the local healthcare system and clinical practices to evaluate values for reimbursement and pricing. Therefore, Statistics and data science have opportunities for contributing toward presenting new methodologies for the RWD.

## Conclusion

The RWD is not new, but rapidly evolving in terms of data source, digital devices for data collection, application fields, and regulations. Statistics and data science should be updated corresponding to these rapid changes for various sources and settings in the RWD. We emphasize on improving methodologies for explaining relevance of the obtained evidence including biases and uncertainty.

## Data Availability

No data was generated and analyzed.
